# Evaluation of classical precipitation descriptions for $\gamma ^{\prime \prime } \,(\hbox {Ni}_{3}\hbox {Nb}{-}\hbox {D0}_{22})$ in Ni-base superalloys

**DOI:** 10.1007/s10853-017-1091-9

**Published:** 2017-04-24

**Authors:** I. J. Moore, M. G. Burke, N. T. Nuhfer, E. J. Palmiere

**Affiliations:** 1 Department of Materials Science and Engineering, The University of Sheffield, Sir Robert Hadfield Building, Mappin Street, Sheffield, S1 3JD UK; 2 Materials Performance Centre, The University of Manchester, The Mill, Sackville Street, Manchester, M13 9PL UK; 3 Department of Materials Science and Engineering, Carnegie-Mellon University, Pittsburgh, PA 15213 USA

## Abstract

The growth/coarsening kinetics of $\gamma ^{\prime \prime }\, (\hbox {Ni}_{3}\hbox {Nb}{-}\hbox {D0}_{22})$ precipitates have been found by numerous researchers to show an apparent correspondence with the classical (Ostwald ripening) equation outlined by Lifshitz, Slyozov and (separately) Wagner for a diffusion controlled regime. Nevertheless, a significant disparity between the actual precipitate size distribution shape and that predicted by LSW is frequently observed in the interpretation of these results, the origin of which is unclear. Analysis of the literature indicates one likely cause for this deviation from LSW for $\gamma ^{\prime \prime }$ precipitates is the “encounter” phenomenon described by Davies et al. (Acta Metall 28(2):179–189, 1980) that is associated with secondary phases comprising a high volume fraction. Consequently, the distributions of both $\gamma ^{\prime \prime }$ precipitates described in the literature (Alloy 718) and measured in this research in Alloy 625 are analysed through employing the Lifshitz–Slyozov-Encounter-Modified (LSEM) formulation (created by Davies et al.). The results of the LSEM analysis show good far better agreement than LSW with experimental distributions after the application of a necessary correction for what is termed in this research as “directional encounter”. Moreover, the activation energy for $\gamma ^{\prime \prime }$ coarsening in Alloy 625 shows conformity with literature data once the effect of heterogeneous (on dislocations) precipitate nucleation at higher temperatures is accounted for.

## Introduction

Since its inception in 1961, the cube root law developed on either side of the iron curtain by Lifshitz, Slyozov [13] and Wagner [29] (LSW) to quantitatively describe the phenomenon of spherical particle coarsening (Ostwald ripening), has been successfully applied to several systems [9]. Furthermore, using an adaptation defined by Boyd et al. [2] a decade later, the LSW approach has been shown to yield an apparently good agreement with experimental measurements of the change in size of non-spherical particles such as $\gamma ^{\prime \prime }$ (Ni$_{3}$Nb–D$0_{22}$) in Ni-base alloys [4, 7, 8, 11, 23, 28, 30, 31]. Despite this apparent success, however, closer scrutiny of the results to which the latter formalism has been applied (such a coarsening of $\theta ^{\prime \prime }$ precipitates in Al–Cu alloys by Boyd et al. [2] in the derivation paper) reveals a marked discrepancy between the real and calculated precipitate size distributions.

Considering the distribution of precipitates like $\gamma ^{\prime \prime }$ particles in Ni-base alloys and $\theta ^{\prime \prime }$ particles in Al–Cu, an immediate source for their distributions deviating from LSW can be identified as their high volume fraction, *viz.* a small fraction (defined such that particle spacing >> particle dimensions) is necessitated by the LSW mechanism. To this end, it is clear that a more accurate description of the coarsening behaviour of these spheroidal precipitates can only be achieved through the use of one of the many different adaptations to the LSW formalism to describe the evolution of precipitates comprising a high volume fraction [1].

Amongst the afore alluded to descriptions, almost all are based principally on approximate solutions/modifications to the solute diffusion description in LSW and, therefore, often require assumptions which are seemingly contradicted by experimental observations. In contrast, the Lifshitz–Slyozov-Encounter-Modified (LSEM) theory of Davies et al. [6] is physically sound owing to its derivation being predicated on the mechanism of precipitate “encounter”; the broadening of the precipitate distribution from the classical LSW shape occurs by the coalescence of precipitates whose diffusion fields have overlapped, i.e. they are said to have “encountered” one another. Such behaviour has been observed for $\gamma ^{\prime \prime }$ precipitates in a number of studies [11, 23, 26].

The governing precipitate growth/coarsening equation originally composed by Davies et al. is strictly applicable only to spherical precipitates but, following the work of Boyd et al. [2] (in their original adaptation to the LSW model), an LSEM cube root law defining the growth of spheroidal particles can be composed according to Eq. 1 where, $\bar{L}_{\text {M}}$ is the average major axis of ellipsoidal particles initially (0) and at time *t*, $X^e$ is the equilibrium solute concentration in the matrix, $\zeta$ is the precipitate–matrix interfacial energy, $V_\text {m}$ is the molar volume of the precipitate, $\alpha$ is the precipitate aspect ratio (minor axis length/major axis length), *R* is the ideal gas constant, *D* is the diffusion coefficient of the solute atoms in the matrix, *T* is the absolute temperature, and $f_{\mathrm{LSEM}}$ and $C_{\text {LSEM}}$ are a function and system constant, respectively, defined by Davies et al. [6]. As a result, it is evident that a more appropriate LSW description of the evolution of the size distribution for high volume fraction ellipsoidal precipitates, accounting for “encounter”, should be possible through LSEM.

1 $$\begin{aligned} {\bar{L}_{\text {M}}^3}(t) - {\bar{L}_{\text {M}}^3}(0) = \frac{32D\zeta V_{\text{m}} X_\gamma^{\text {e}}t }{\alpha \pi RT } \frac{f_{\text {LSEM}}(\bar{L}_{\text {M}}, L_{\text {M}}^*)^3}{C_{\text {LSEM}}} \end{aligned}$$

Owing to the importance of the precipitate population in determining the mechanical properties of the superalloys containing $\gamma ^{\prime \prime }$ [15, 16], and in light of the aforementioned likely increase in the accuracy of the replicated size distribution that should result from adopting the LSEM theory *cf.* LSW, the principle aim of this work was to assess the evolution of $\gamma ^{\prime \prime }$ populations with respect to LSEM. In the absence of any adaptation being made to the LSEM theory, a verbatim repeat of its specific intricacies as discussed by Davies et al. [6] is avoided.

## Literature Alloy 718 $\gamma ^{\prime \prime }$ distributions

The study of $\gamma ^{\prime \prime }$ precipitate evolution with respect to LSW evolution kinetics has been most commonly investigated in Alloy 718, but only a handful of relevant studies report on the precipitate size distribution (PSD) [8, 11, 28]. As a result, given that analysis of the PSD shape constituted the raison d’être of this investigation it was purely the analysis of Han et al. [11], Sundararaman et al. [28] and Dong et al. [8] that formed the basis of the application of the LSEM theory to previously published $\gamma ^{\prime \prime }$ precipitation studies here.

### Volume fraction

**Figure 1:**
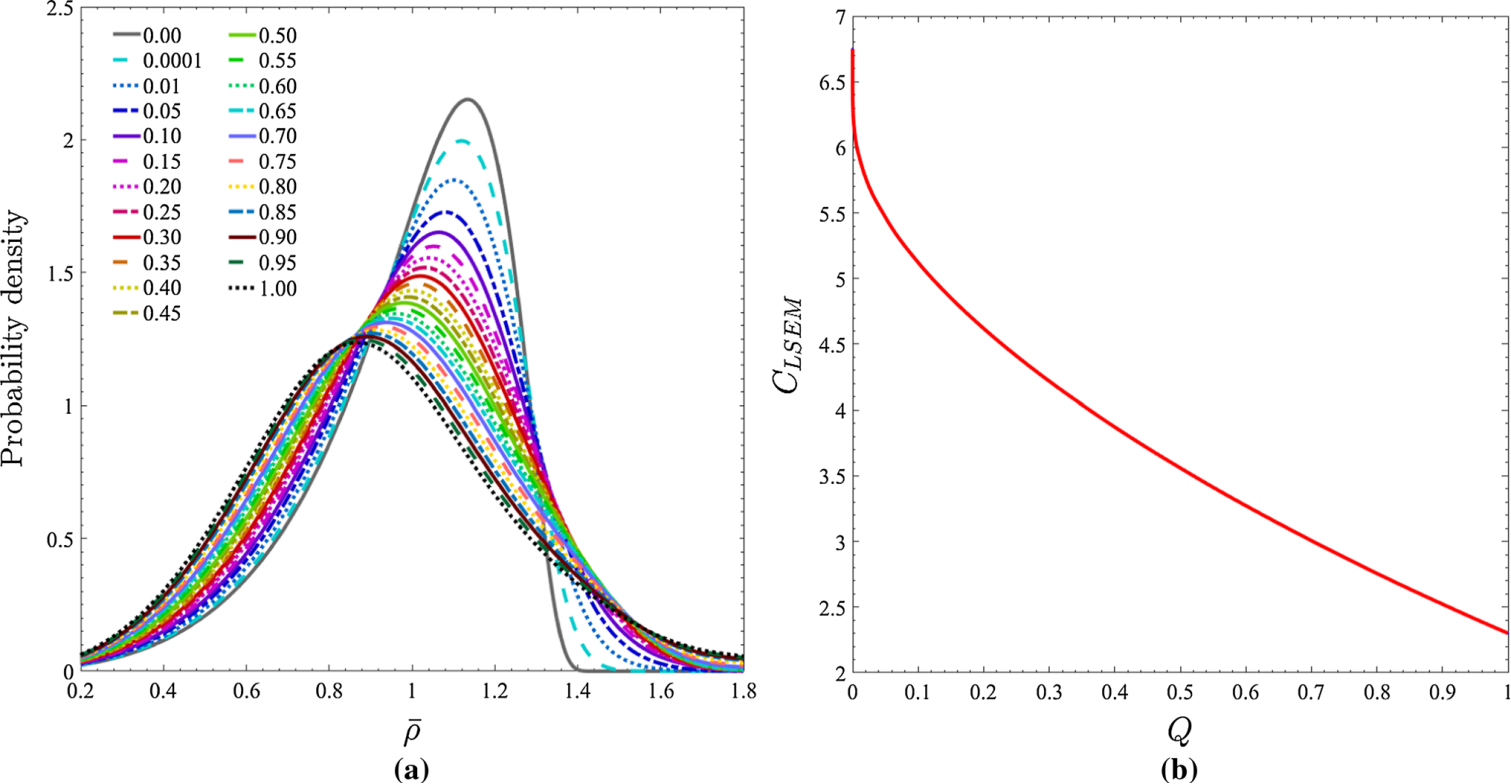
**a** LSEM distributions corresponding to different precipitate volume fractions *Q* where $\bar{\rho }$ is average value of a precipitate size dimension. **b** $C_{\text {LSEM}}$ behaviour with *Q* calculated by Davies et al. [6]

As presented in Fig. 1, both the LSEM probability density and $C_{\text {LSEM}}$ depend crucially on the precipitate volume fraction *Q*. Unfortunately, however, neither Han et al. citeHan, Sundararaman et al. [28] nor Dong et al. [8] provide a direct quantification of *Q* in their studies. In general, this eventuality would present a significant problem as the separate calculation of *Q* would only be possible through extensive precipitation kinetics computations but in the case of high ($\gtrapprox$10%) volume fraction precipitates such as $\gamma ^{\prime \prime }$, the sensitivity of the LSEM probability density (Fig. 1a) is such that an approximation is instead reasonable. Explicitly, the precipitate volume fraction can be set equivalent to one in which all of the niobium is contained within $\gamma ^{\prime \prime }$ precipitates. Consequently, assuming a stoichiometric Ni$_{3}$Nb composition, the value of *Q* for $\gamma ^{\prime \prime }$ in each Alloy can be calculated from their respective compositions (at.%) listed in Table 1 such that $Q = 4\,\times$ Nb(at.%).

**Table 1:** Compositions (at.%) of the Alloy 718 samples reported in the cited studies

Study	Ni	Cr	Fe	Mo	Nb	C	Mn	Si	S	Al	Ti	B	Cu
Dong [8]	$50.65^*$	21.06	19.89	1.82	2.93	0.10	$0^+$	$0^+$	$0^+$	2.15	1.39	0.02	$0^+$
Han [11]	$51.62^*$	20.26	19.90	1.85	3.07	0.14	0.20	0.62	0.01	1.11	1.17	$0^+$	0.04
Sundararaman [28]	$51.78^*$	20.39	18.66	1.75	3.73	0.19	0.22	0.60	$0^+$	2.14	0.54	$0^+$	$0^+$

The indexes $^*$ and $^+$, respectively, signify the quantity is calculated from balance or was not indicated

#### LSEM results

**Figure 2:**
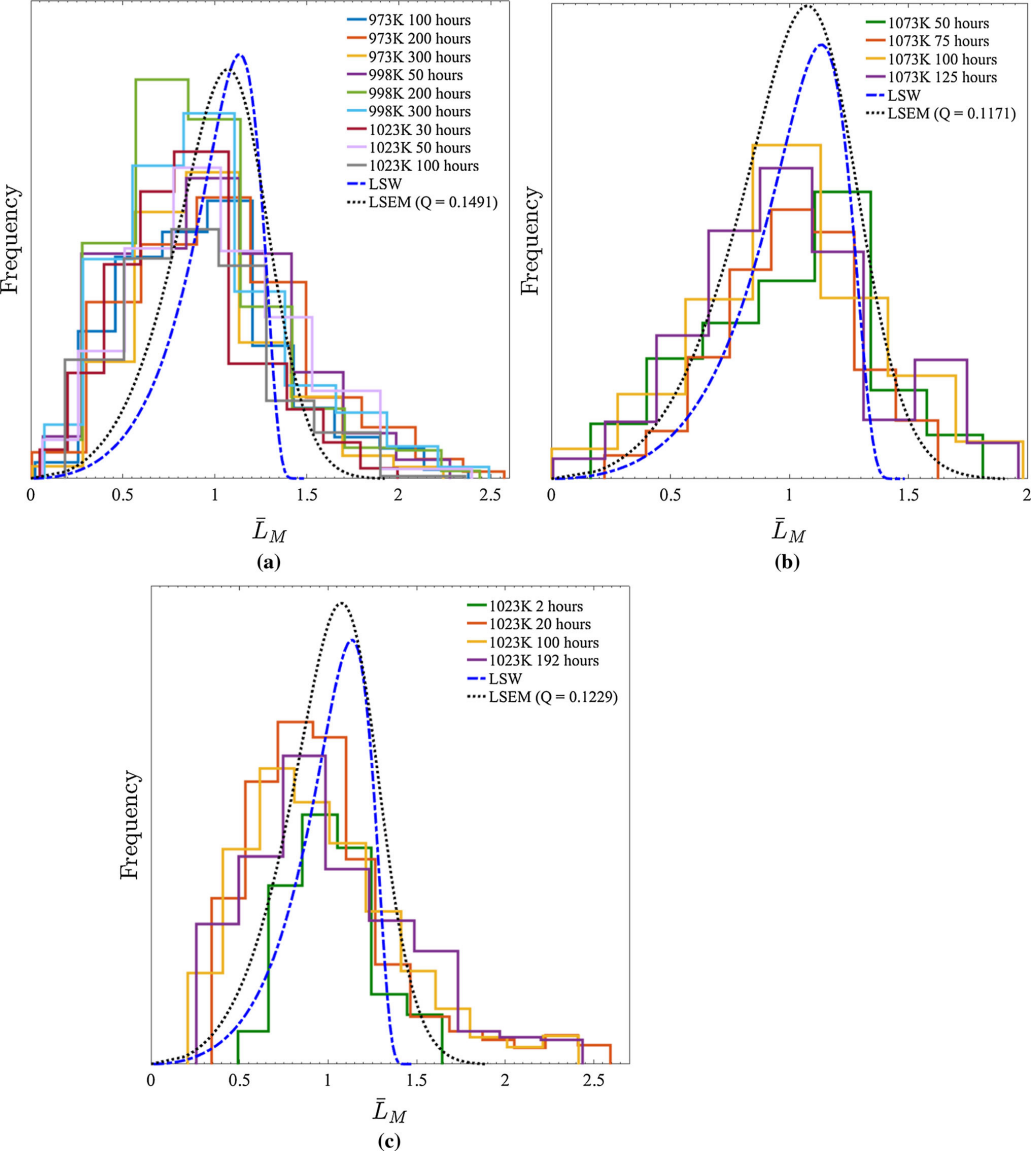
Original precipitate size distributions measured by **a** Han et al. [11], **b** Dong et al. [8] and **c** Sundararaman et al. [28] in material (compositions in Table 1) aged at for the labelled conditions. LSW and LSEM distributions are also illustrated, with the corresponding *Q* values for the latter indicated. Owing to the specific focus on the shape of the precipitate size distributions, for ease of comparison each has been normalised to the same relative classified magnitude meaning their specific values are arbitrary

The LSEM and LSW distribution profiles computed for the three Alloy 718 studies of Han et al. [11], Sundararaman et al. [28] and Dong et al. [8], as well as the originally published PSDs, are presented in Fig. 2.^1^ In each instance, it is evident that the LSEM curves bare a much closer resemblance (*cf. LSW*) to the experimental data (particularly the measurements by Dong et al. [8] for precipitate populations existing after 100 h at 1073 K); however, there still remains a marked discrepancy. As a result of this outcome, whilst the LSEM description can be concluded as a far more appropriate description for $\gamma ^{\prime \prime }$ precipitation in Alloy 718, it is clear that additional modifications to the application of the theory are required.

### Correction to LSEM for $\gamma ^{\prime \prime }$ “Directional Encounter”

**Figure 3:**
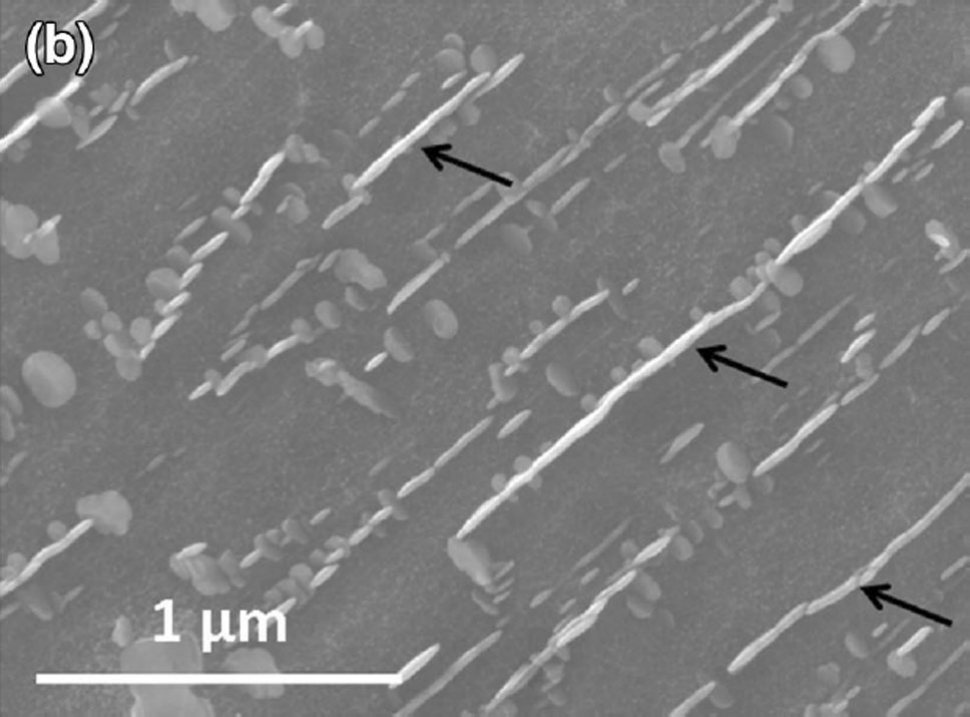
Secondary electron (SE) image obtained by Suave et al. [24] of extensive, large scale $\gamma ^{\prime \prime }$ in a sample of Alloy 625. A tendency for precipitates to agglomerate along their major axes and for the number of agglomerated precipitates to increase with resolvable precipitate size is clearly discernible. The results of Han et al. [11] indicate that this is not related to imaging or etching effects

As depicted most strikingly in the literature by Suave et al. [24] (image reproduced for ease of reference in Fig. 3), rather than the isotropic agglomeration behaviour mathematically described by Davies et al. $\gamma ^{\prime \prime }$ precipitates are observed to coalesce though a directional mechanism (in that they seemingly almost always coalesce through conjunction along their major axis) referred to here on as “directional encounter”.

Considering the variables governing precipitate evolution it is clear that, given the continuity of the material condition, the principle cause of “directional encounter” can only be assigned to the varying misfit strains surrounding the precipitates: $\gamma ^{\prime \prime }$ has been shown to possesses a high coherency with the precipitate matrix, however its body-centre-tetragonal (BCT) structure leads to a significant variation in the misfit strain surrounding the precipitate [16]. Furthermore, as demonstrated from the results of Slama et al. presented in Table 2, the level of misfit strain (calculated according to the strain tensor defined by Cozar et al. [5]) along the two principle axes of a precipitate is observed to follow opposite trends as the shape change discussed previously proceeds. Consequently, with smaller strain posing a lower energy barrier to precipitate “encounter”, it is evident that coalescing precipitates will tend to not only join along their major axis but will do this with increasing propensity as they grow owing to the lower, and continually decreasing value, of the ratio $\epsilon _{11}/\epsilon _{33}$.

**Table 2:** Average values of the aspect ratio ($\bar{\alpha }$) and the FCC-BCT strain tensor components $\epsilon _{11}$ and $\epsilon _{33}$ for $\gamma ^{\prime \prime }$ precipitates in Alloy 718 as measured by Slama et al. [23]

		Temperature (K)					
		953			1023		
		$\epsilon _{11}$	$\epsilon _{33}$	$\bar{\alpha }$	$\epsilon _{11}$	$\epsilon _{33}$	$\bar{\alpha }$
Ageing time (h)	4	0.0080	0.0303	−	0.0089	0.0296	0.300
	50	0.0042	0.0323	0.333	0.0036	0.0321	0.187
	98	0.0033	0.0377	0.181	0.0030	0.0331	0.178

**Figure 4:**
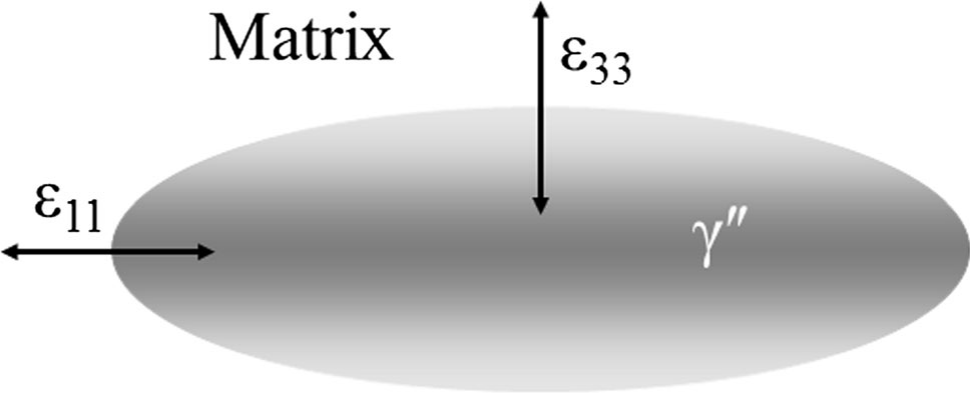
Directions of the strain tensor components $\epsilon _{11}$ and $\epsilon _{33}$ with respect to the oblate spheroidal shape of $\gamma ^{\prime \prime }$ precipitates

The directions of tensor components with respect to the precipitate are shown in Fig. 4

**Figure 5:**
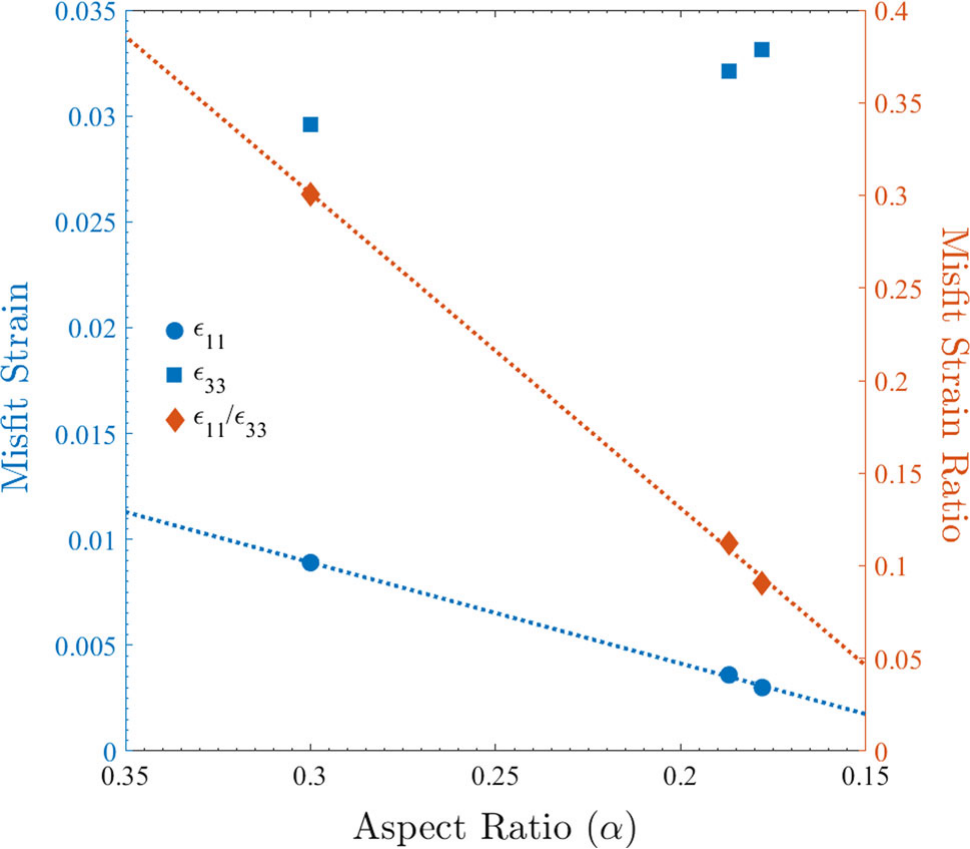
Evolution of the average value of the strain tensor components $\epsilon _{11}$ and $\epsilon _{33}$, and separately their ratio, with the average aspect ratio of $\gamma ^{\prime \prime }$ precipitates measured by Slama et al. [23] in Alloy 718 aged at 1023 K. Linear fits to $\epsilon _{11}$ and the ratio are also indicated

In light of the aforementioned origin for “directional encounter”, it is obvious that the phenomenon will principally act to inflate the values of $L_{\text {M}}$ and, thus, that application of LSEM to $\gamma ^{\prime \prime }$ precipitates would be more appropriately made to experimental curves where the effect of increased “encounter” probability with precipitate size has been removed. Accordingly, harnessing the fact that the enhancement probability correlates directly to both the relative and absolute values of $\epsilon _{11}$ and $\epsilon _{33}$, an evaluation of its behaviour with $\alpha$ can be determined through application of the results of Slama et al. at 1023 K in Fig. 5. Specifically, there are three important facts: (1) $\epsilon _{11}$ evolves apparently linearly with respect to $\alpha$, (2) the evolution of the ratio $\epsilon _{11}{:}\epsilon _{33}$ is also well approximated by a linear relationship with respect to $\alpha$ and (3) the probability of any two precipitates agglomerating and the $\alpha$ of the particle they form are proportional to the average of their aspect ratios, the inflation of a specific precipitate size in the distribution can be quantified as proportional to $\alpha ^{-1}$ by the mechanism outlined previously. Consequently, in view of the exact relationship being unknown, the conversion of the original $L_{\text {M}}$ distribution to one compatible with LSEM can be performed to a reasonable first approximation through the multiplication of $L_{\text {M}}$ by the magnitude of $\alpha (L_{\text {M}})$, i.e. $f(L_{\text {M}}) \rightarrow f(L_{\text {M}} \times \alpha (L_{\text {M}}))$.

### Shape change

**Figure 6:**
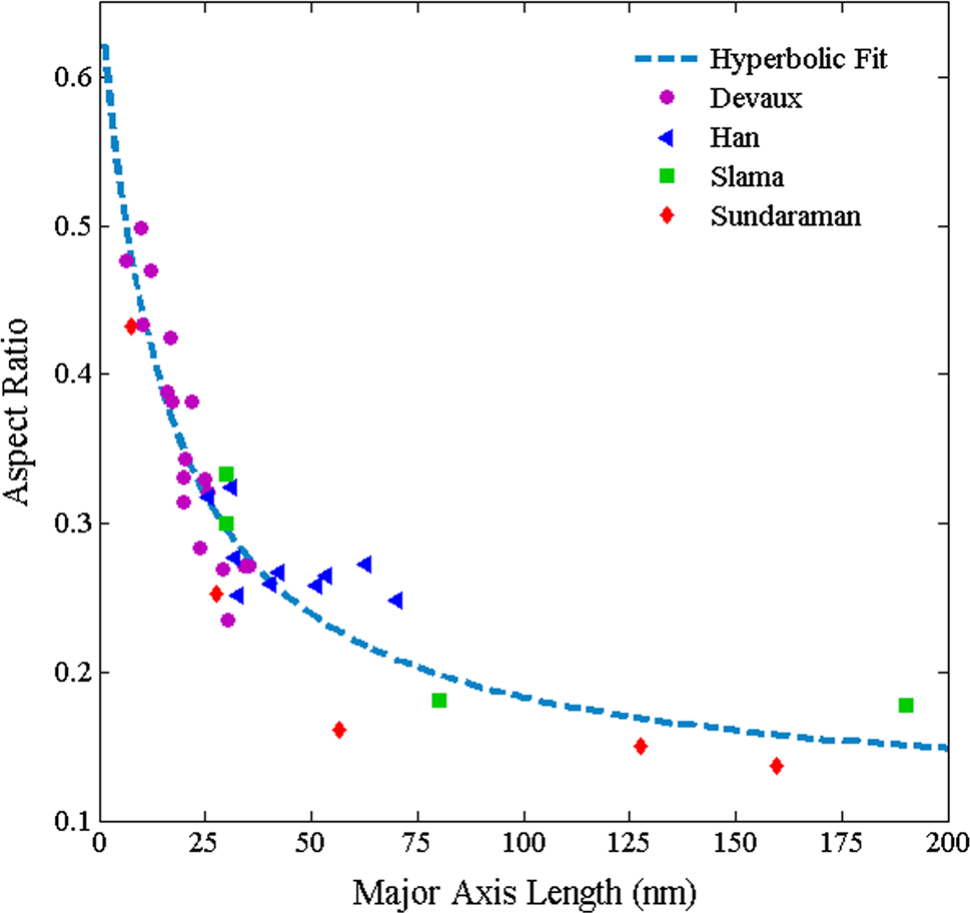
Alloy 718 $\gamma ^{\prime \prime }$ aspect ratio ($\alpha$) versus major axis length plot constructed by Devaux et al. [7] based on measurements made on their own samples and literature data (Han [11], Slama [23], Sundararaman [28]). Hyperbolic fit made to the data for calculating aspect ratios in the present research for the precipitates contained within the PSDs of Han et al. [11] and Dong et al. [8] is indicated

Fundamental to the previously described mechanism accounting for “directional encounter” is the evaluation of the function $\alpha (L_{\text {M}})$. In this regard, the shape evolution behaviour of $\gamma ^{\prime \prime }$ analysed most comprehensively in the literature by Devaux et al. [7], through the comparison of their own results with others published in the literature (presented in Fig. 6), is utilised. Specifically, following the hyperbolic relationship demonstrated for this parameter in Alloy 625 by the present authors elsewhere [14], the behaviour of $\alpha (L_{\text {M}})$ is defined according to a hyperbolic decay curve fitted to values of $\bar{\alpha }(L_{\text {M}})$ measured by Devaux et al. and others. The use of separate fits for each dataset is precluded because the small number of data points and/or their relative narrow range with respect to $L_{\text {M}}(t)$, despite the possible influences from temperature and composition differences. However, that being stated, it should be pointed out that owing to the specific magnitude of the correction applied (explicitly that it essentially converts the major axis values in to minor axis values) the fact that Sundararaman et al. [28] also provided a quantification with respect to the minor axis distribution means that the application of the function is not needed to their data, *viz.* this latter distribution set can be harnessed instead.

#### Results

**Figure 7:**
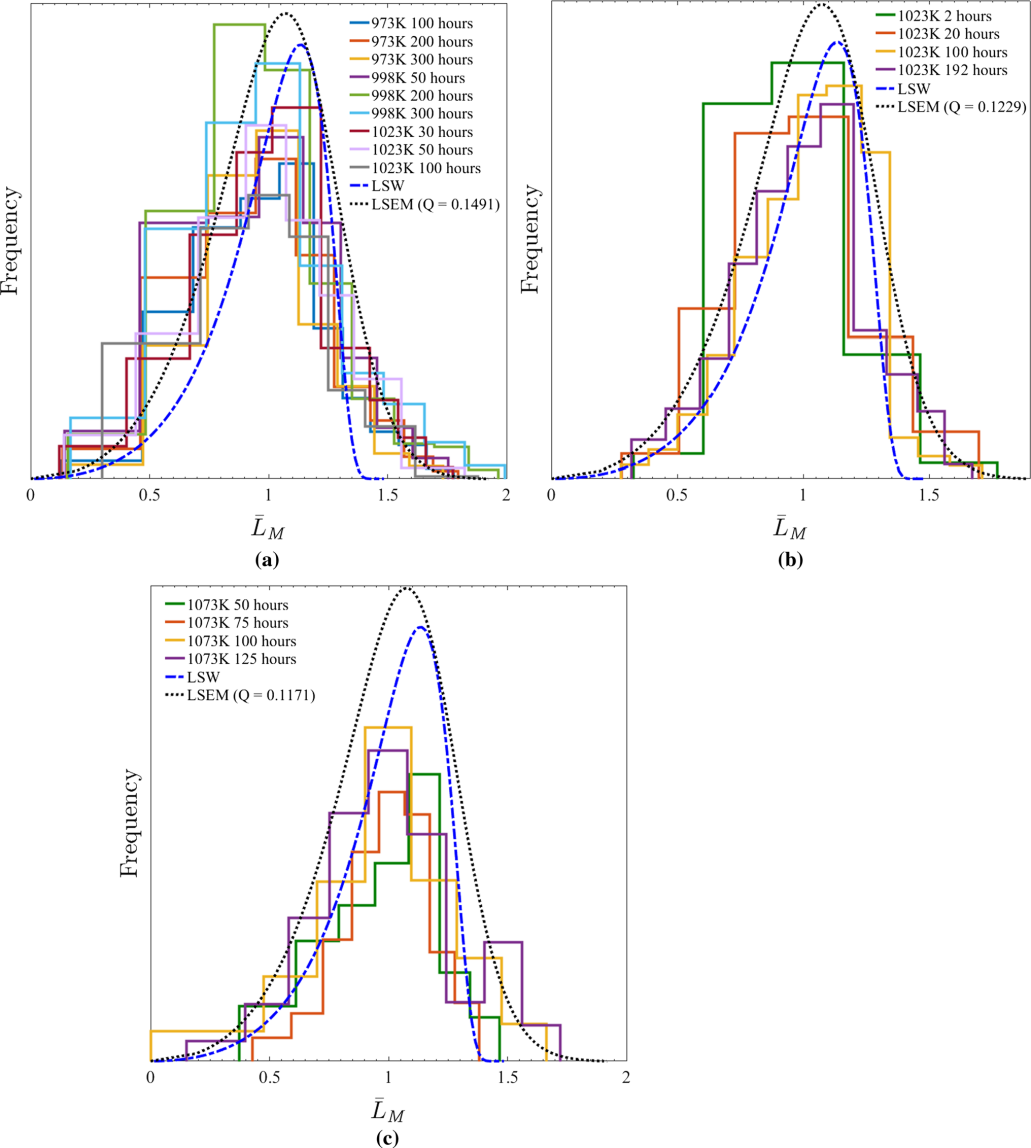
Precipitate size distributions (corrected for “directional encounter”) measured by **a** Han et al. [11] and **b** Dong et al. [8]. **c** $f(L_{\text {M}} \times \alpha (L_{\text {M}}))$ distribution measured by Sundararaman et al. [28]. Material (composition in Table 1) aged according to the correspondingly labelled conditions. LSW and LSEM distributions are also illustrated, with the corresponding *Q* values for the latter indicated

Employing the predefined methodology, the LSEM distribution profiles and “directional encounter” corrected experimental PSDs computed for the three Alloy 718 studies of Han et al. [11] and Dong et al. [8] are presented in Fig. 7a, b (see footnote 1). Additionally, the minor axis precipitate distribution measured by Sundararaman et al. [28], equivalent to $f(L_{\text {M}} \times \alpha (L_{\text {M}}))$ is shown in Fig. 7c. Inspection of each distribution set reveals that the modification for “directional encounter” yields a much closer agreement with the both the LSEM and LSW curves; however, the level and particular type of correspondence varies between studies and ageing conditions: all of the PSDs published by Han et al. [11] (Fig. 7a) display a consistent and strikingly good conformity with the constructed LSEM distribution except those measured from precipitates in samples aged at 998 K for durations $\ge 200$ h as both retain a noticeable skew. Similarly, the PSDs measured by Sundararaman et al. [28] also show a generally good agreement with LSEM compared to LSW but the specific the shape and therefore level of agreement changes between curves.

In contrast to the other two studies, the curves of Dong et al. [8] highlight a transitional behaviour such that the curves for populations formed at times $\le 75$ h agree strongly with LSW, whereas populations measured after 100 h of ageing conform with LSEM. Nevertheless, all of the curves can be seen to be far better parametrised by the respective mathematical distributions after the implementation of the “directional encounter” correction.

Considering the results of Han et al. [11] first, the most obvious cause for the retention of the skew in the experimental data at 998 K can be simply determined as the fit in Fig. 6. Firstly, the necessary (due to the limited range and/or the sparsity of data points in any one study) use of results obtained from different alloys, with different compositions and aged at different temperatures, means the $\alpha (L_{\text {M}})$ trend is likely inaccurate for any one specific alloy and, therefore, leads to an error in the calculation pertaining to the removal of the “directional encounter” enhancement. Secondly, as “directional encounter” results in a consistent decrease in $\alpha$ at large precipitate radii, the assumption of a hyperbolic relationship is likely not valid at larger $L_{\text {M}}$ values, *viz.* the value of $\alpha$ used in the “directional encounter” correction is overestimated in some instances, particularly the highlighted conditions used by Han et al. [11] which apparently yielded the largest precipitates. This latter result is supported by the $\alpha (L_{\text {M}})$ trends presented recently by Zhang et al. [31] which show a continued, significant reduction in $\alpha$ when $L_{\text {M}}$ > 200 nm.

Turning to the measurements of Dong et al., the stronger agreement with the LSEM distribution at long ageing times and with the LSW distribution at short durations can be attributed to the specific nature of the $\gamma ^{\prime \prime }$ precipitation observed in the alloy: As described by Dong et al., their modified Alloy 718 composition produced a compact $\gamma ^{\prime }$–$\gamma ^{\prime \prime }$ precipitate morphology. Consequently, it is straightforward to conclude that at the start of ageing the inhibiting influence of $\gamma ^{\prime }$ reduced general precipitate “encounter” such that (aside from the “directional encounter” mechanism) an almost purely diffusion based evolution took place. Similarly, whilst “encounter” still occurred, the higher probability for large $\gamma ^{\prime \prime }$ precipitates to be restricted by $\gamma ^{\prime }$ suppressed their agglomeration to a value lower than it otherwise would have been. This second phenomena are actually evidenced in Fig. 7 by the “over correction” of larger precipitate sizes in the more extensively aged material to values below that of the LSEM distribution but above LSW.

## Alloy 625 $\gamma ^{\prime \prime }$ distributions

In light of the above results for Alloy 718, the correction devised and implemented for “directional encounter” can be seen to result in a far better adherence of measured $\gamma ^{\prime \prime }$ precipitate statistics to classical nucleation, growth and coarsening descriptions. Moreover, the LSEM theory can be concluded as a more accurate representation of $\gamma ^{\prime \prime }$ than LSW. In this regard, therefore, an analysis of $\gamma ^{\prime \prime }$ precipitation in Alloy 625 was undertaken, utilising the correction for “directional encounter” in all instances.

### Experimental details

Subsequent to a solution anneal at 1423 K for 30 min to remove any previously existing precipitates (following the successful demonstration by Shankar et al. [21] of such a treatment), Alloy 625 specimens (cut from a bar with the composition detailed in Table 3) were aged, based on the TTT curve published by Floreen et al. [10], at temperatures between 873 and 1023 K for durations of up to 3000 h. Quantification of the precipitate population was made through analysis of <100> centred dark-field transmission electron microscopy (TEM) images obtained from electron-transparent specimens prepared through twin-jet electropolishing (using a 20% $\hbox {HClO}_4$ 80% $\hbox {CH}_{3}\hbox {OH}$ electrolyte) 3 mm discs of material mechanically thinned to a thickness of $<100\, \mu \hbox {m}$.

**Table 3:** Composition (at.%) of the Alloy 625 samples in analysed in this work

Ni	Cr	Fe	Mo	Nb	C	Mn	Si	P	S	Al	Ti
$63.05^*$	24.85	3.05	5.32	2.43	0.10	0.22	0.49	0.02	0.01	0.26	0.20

The index $^*$ signifies the quantity is calculated from balance

### Results

#### Evidence for “directional encounter”

**Figure 8:**
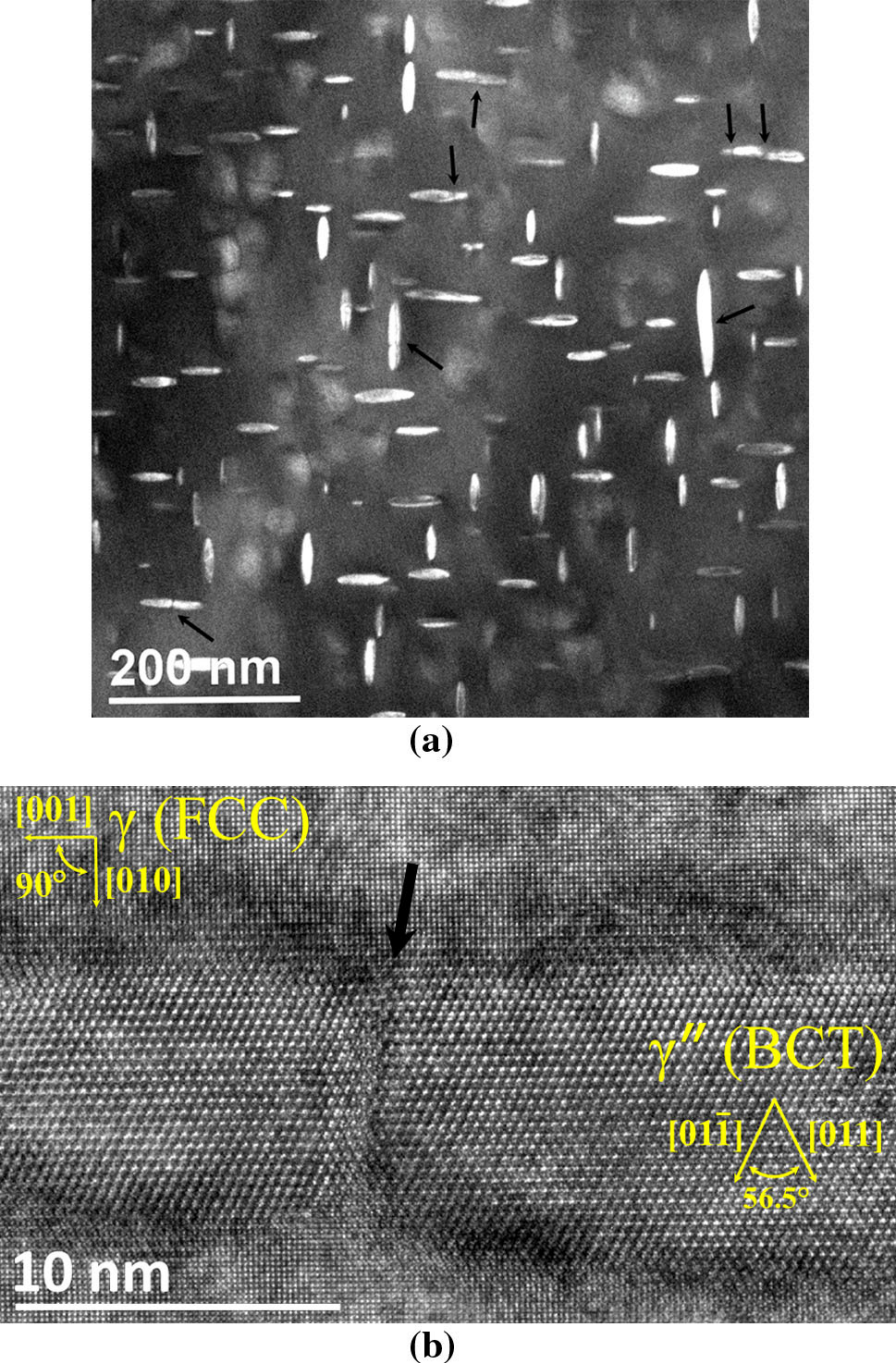
**a** [001] Oriented dark-field TEM image showing “coalescing” $\gamma ^{\prime \prime }$ precipitates (*black arrows*) and **b** [100] oriented high-resolution-TEM (HRTEM) image of two coalescing $\gamma ^{\prime \prime }$ precipitates, both in Alloy 625 aged at 923 K. The crystal structures of both the matrix (FCC) and precipitate (BCT) phases are clearly visible in image **b** indicating the presence of a “defect-like” region (*arrowed*) between the two BCT precipitate lattices at the join along their major axes

An [001] oriented dark-field TEM image and a HRTEM image showing the crystal structures of two $\gamma ^{\prime \prime }$ precipitates that appear to have “coalesced” along their major axes in Alloy 625 aged at 923 K is presented in Fig. 8. A high degree of coherency/lattice matching at the interface between the $\gamma ^{\prime \prime }$ and FCC ($\gamma$) matrix is clearly evident, as these precipitates exhibit a well-defined orientation relationship with the matrix. In addition, this particular $\gamma ^{\prime \prime }$ exhibits an apparent “defect” region at the location of the “coalescence” of two discrete precipitates, strongly suggesting an agglomeration process rather than simple impingement has occurred; the presence of dislocations indicates that there is some degree of coherency and that the coalescence of the two precipitates is not complete. This result constitutes a significant progression on the tilting analysis of Han et al. [11] in Alloy 718 by providing direct evidence of the agglomeration process.

#### $$\gamma ^{\prime \prime }$$$$\alpha (L_{\text {M}})$$

**Figure 9:**
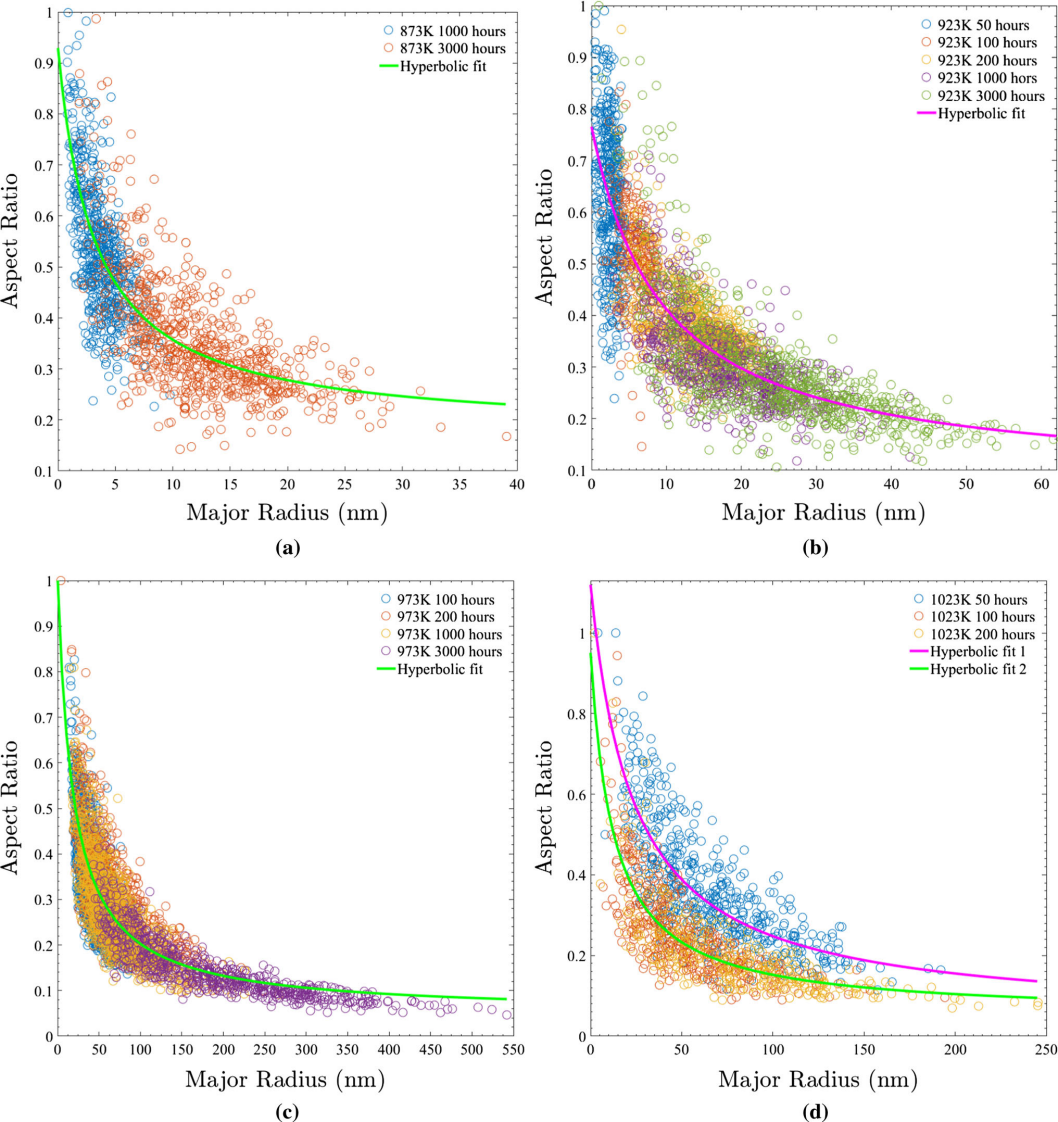
$\gamma ^{\prime \prime }$ aspect ratio versus major axis length plot created from populations formed when ageing at **a** 873 K, **b** 923 K, **c** 973 K and **d** 1023 K

In contrast to the approach taken with respect to Alloy 718 previously, the acquisition of data from a large number (100–1000 s) of precipitates in each aged Alloy 625 sample facilitated the calculation of separate (isothermal) mathematical fits to define $\alpha (L_{\text {M}})$ as shown in Fig. 9. Leaving aside the two population behaviour indicated at 1023K, as this will be discussed later, and accepting these fits as sufficient for the calculation of the distributions with respect to $({\bar{L}}_{\text {M}}$, the lack of comparable data sets both with respect to precipitate number and temperature variation in the literature means it is worth remarking upon the specific behaviour exhibited.

Significantly, the separate isothermal plots for $\alpha (L_{\text {M}})$ in Fig. 9 display not only a noticeable difference in the shape-changing behaviour of $\gamma ^{\prime \prime }$ precipitates, but also the existence of a gradual continual evolution in each instance rather than the step change (attributable to a change in coherency) theorised (from experimental observations) by both Cozar et al. [5] and Devaux et al. [7] and the recent modelling work of Ji et al. [12].

The explanation for these observations is the change in physical characteristics of both the precipitate and matrix. As the shape behaviour of $\gamma ^{\prime \prime }$ precipitates is ultimately a consequence of precipitate–matrix strain anisotropy, which changes gradually with temperature [14, 23] different isothermal ageings can exhibit different $\gamma ^{\prime \prime }$ evolution behaviours. Furthermore, the mathematical descriptions developed by Cozar et al. [5] and Ji et al. [12] assume constant values for parameters such as the misfit strain for coherent precipitates in contrast to the measurements of Slama et al. [23] in Table 2, which means that the step change can be more appropriately interpreted as the region where these assumptions completely break down. This fact was accounted for by Ji et al. [12] through their alternate use of two sets of strain values in their calculations: one exclusively for fully coherent precipitates and one exclusively for semi-coherent precipitates. However, the step behaviour they computed (and set out to replicate based on the previous conclusions by Devaux et al. [7]) is clearly still not in agreement with continuous behaviour presented in Fig. 9, signifying that their two-stage behaviour treatment is also too simplistic.

#### $\gamma ^{\prime \prime }$ Distributions

**Figure 10:**
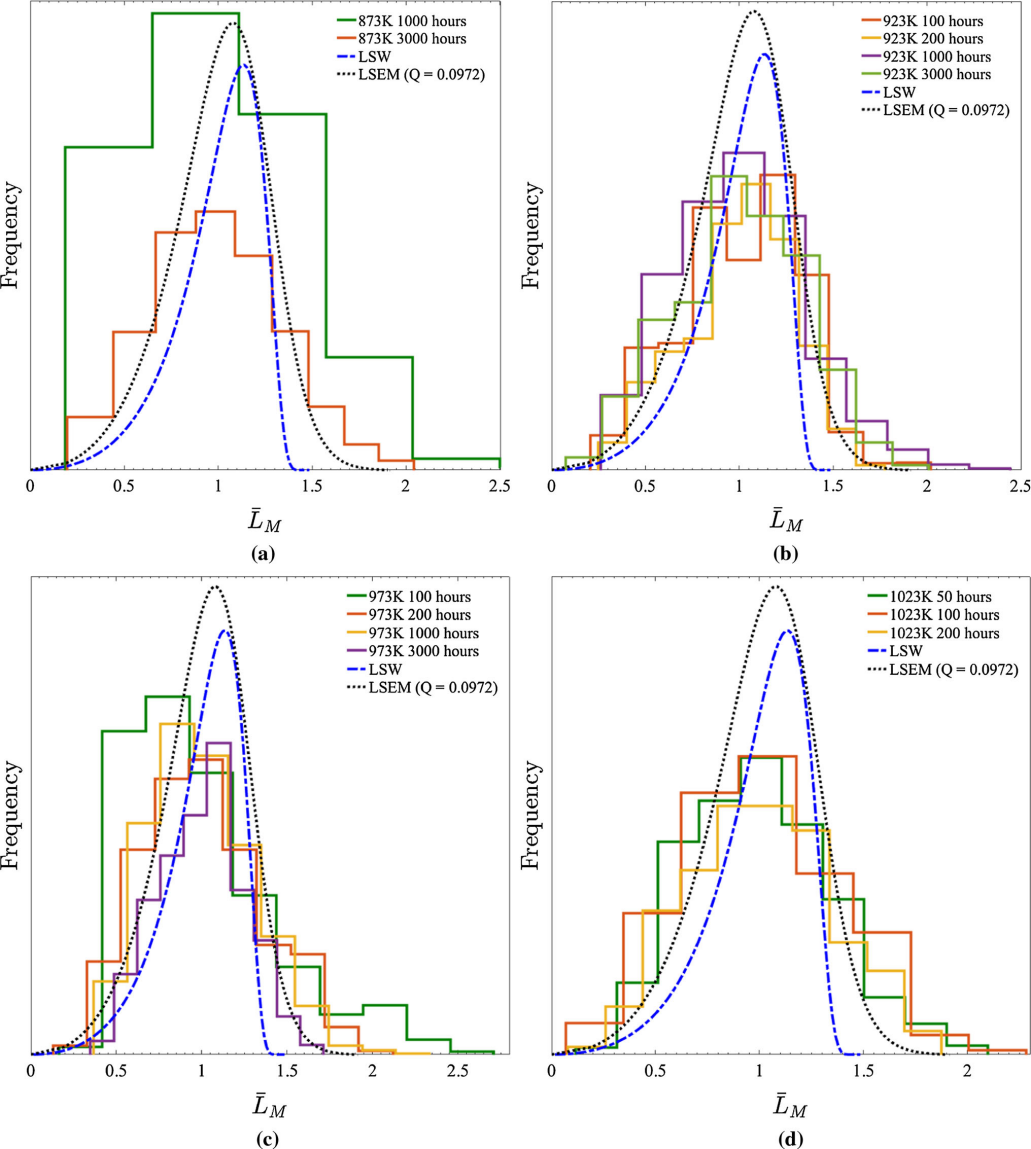
$\gamma ^{\prime \prime }$ precipitate size distributions (corrected for “directional encounter”) measured in Alloy 625 (composition defined in Table 3) aged for the indicated durations at **a** 873 K, **b** 923 K, **c** 973 K and **d** 1023 K. LSW and LSEM distributions corresponding to a *Q* value of 0.0972 in all cases are also illustrated

The “directional encounter” corrected distributions of the Alloy 625 $\gamma ^{\prime \prime }$ precipitates measured in this research are presented, along with the corresponding LSW and LSEM plots, in Fig. 10. Due to the availability of data, the correction was implemented individually to the raw values of $L_{\text {M}}$ using their corresponding $\alpha$ values.

Considering the data pertaining to ageing treatments at 873 and 923 K, it is observed in Fig. 10 that, compared to the previous analysis of Alloy 718 (Fig. 7), the LSEM distributions for $\gamma ^{\prime \prime }$ precipitate more accurately represent those constructed from experimental measurements of populations in Alloy 625. That being said, the curves still display a minor discrepancy because of a remnant skew in the experimental data. Two principle reasons for this discrepancy are identified; (1) the sectioning of particles at the TEM sample surfaces acts to skew the data by concomitantly reducing the number of large particles and inflating the number of smaller ones, and (2) the similar effect of the image analysis technique through sectioning perpendicular particles (as $\gamma ^{\prime \prime }$ precipitates align exclusively along the [001] FCC matrix lattice planes [22]) which have impinged one another owing to their edges being irresolvable. Nonetheless, notwithstanding these aforementioned sources of error (let alone any random sources), the close matching with the LSEM distribution can be interpreted as a success of the correction for “directional encounter”.

**Figure 11:**
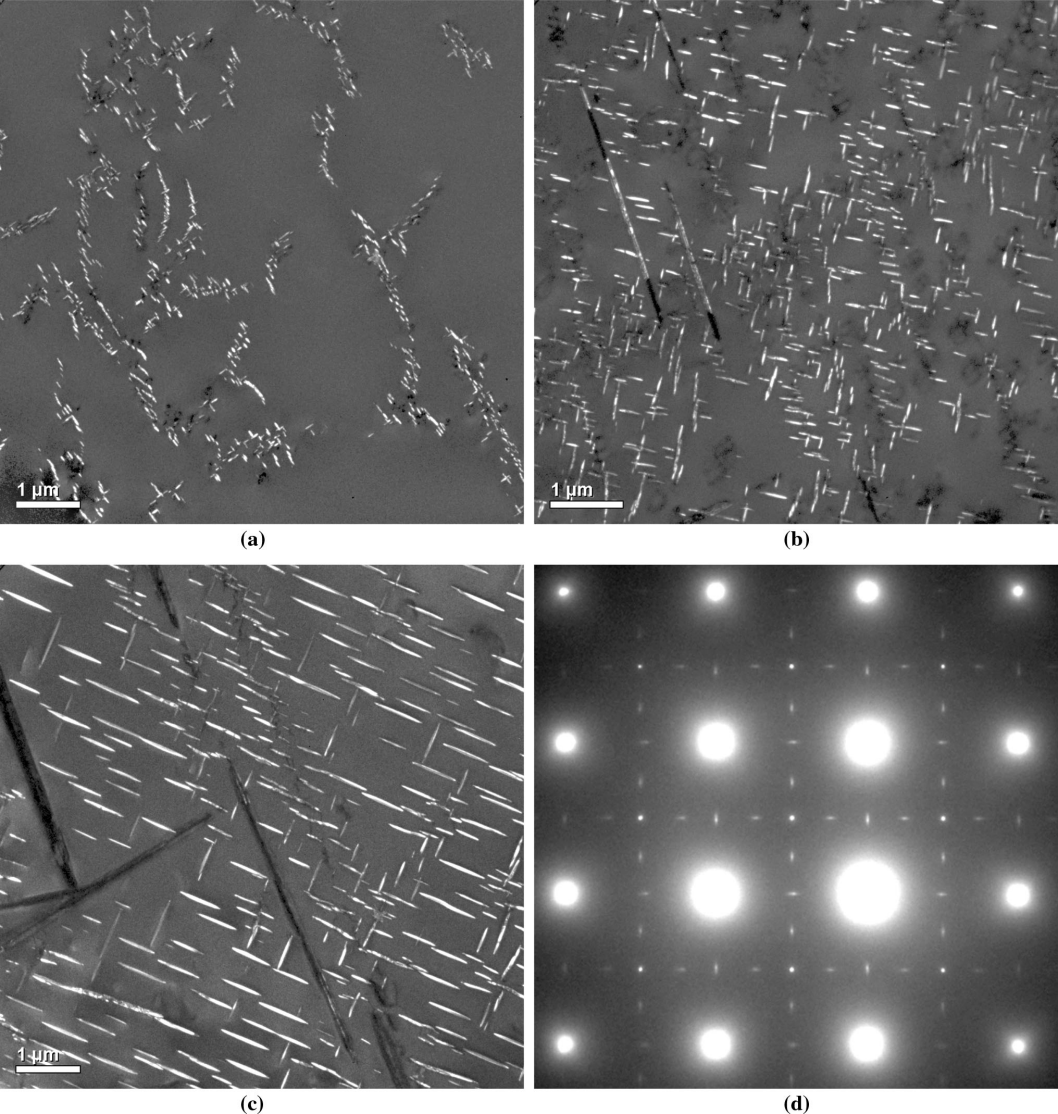
[001]-Oriented *dark-field* TEM image of $\gamma ^{\prime \prime }$ precipitates in samples of Alloy 625 aged at 973 K for **a** 100 h, **b** 1000 h and **c** 3000 h. **d** A [001] selected area electron diffraction pattern showing reflections from the three variants of $\gamma ^{\prime \prime }$, with the streaked precipitate reflections corresponding to the two edge-on variants [16]. A transition from an exclusively heterogeneously nucleated $\gamma ^{\prime \prime }$ population to one comprising both heterogeneously and homogeneously nucleated $\gamma ^{\prime \prime }$ precipitates is clearly visible. Also, needle-like $\delta$ precipitates can be observed in (**c**)

In contrast to the data obtained for lower ageing temperatures, a comparison of the shape LSEM distribution and the precipitate populations formed at 973 and 1023 K reveals a marked disparity; only the populations formed at 973 K after 3000 h and after 200 h at 1023 K show a good correspondence with LSEM. The origin of this discrepancy can be attributed to the fact that at both of these temperatures, rather than a single precipitate population what is actually observed (as shown in Fig. 11) is two separate populations, with one nucleated on dislocations and the other in the matrix. As directly evidenced by the two plots in Fig. 9d, the first population to form preferentially on pre-existing dislocations evolves in a very different manner to those which nucleate homogeneously later in the matrix. Specifically, the different strain environment means the heterogeneously nucleated precipitates do not experience the same forces for shape changing or “directional encounter” and ultimately grow much faster. It is the resultant effect of the statistics of the two precipitate populations being combined on the relative calculated magnitudes of $L_\text {M}$ which lies at the heart of the enhanced skew of the shorter-aged populations in Figs. 10c and d and its reduction in the longer-aged samples thanks to dominance of homogeneously nucleated $\gamma ^{\prime \prime }$ (analogous to those observed at 873 and 923 K).

Taking into account, the present results alone, because of the faster removal/annealing rate of remnant dislocations that should accompany higher temperature ageing, the heterogeneous (on dislocations) to homogeneous nucleation transition behaviour observed could reasonably be considered as related to the Alloy 625 sample thermal history. However, in view of the published literature on similarly aged material it more likely to be attributable to the effect of undercooling as argued by Sundararaman et al. [25, 26] and Pai et al. [17] (and based on the TTT diagrams of Schnabel et al. [19] and Floreen et al., respectively); by increasing the barrier to homogeneous nucleation in a system containing sufficient heterogeneous sites, a reduction in the differential to the solvus temperature will (according to classical theories [18]) ultimately lead to a change in the dominant nucleation site for $\gamma ^{\prime \prime }$ precipitation. Furthermore, if the number of heterogeneous nucleation sites is reduced (through precipitation) by a sufficient amount, the transition between the two mechanisms at the same ageing temperature is reversed [17, 25, 26]. It should be pointed out that both Shaikh et al. [20] and Suave et al. [24] also report on the phenomenon but provide no discussion of the mechanism behind it.

### Activation energy for $\gamma ^{\prime \prime }$ coarsening

**Figure 12:**
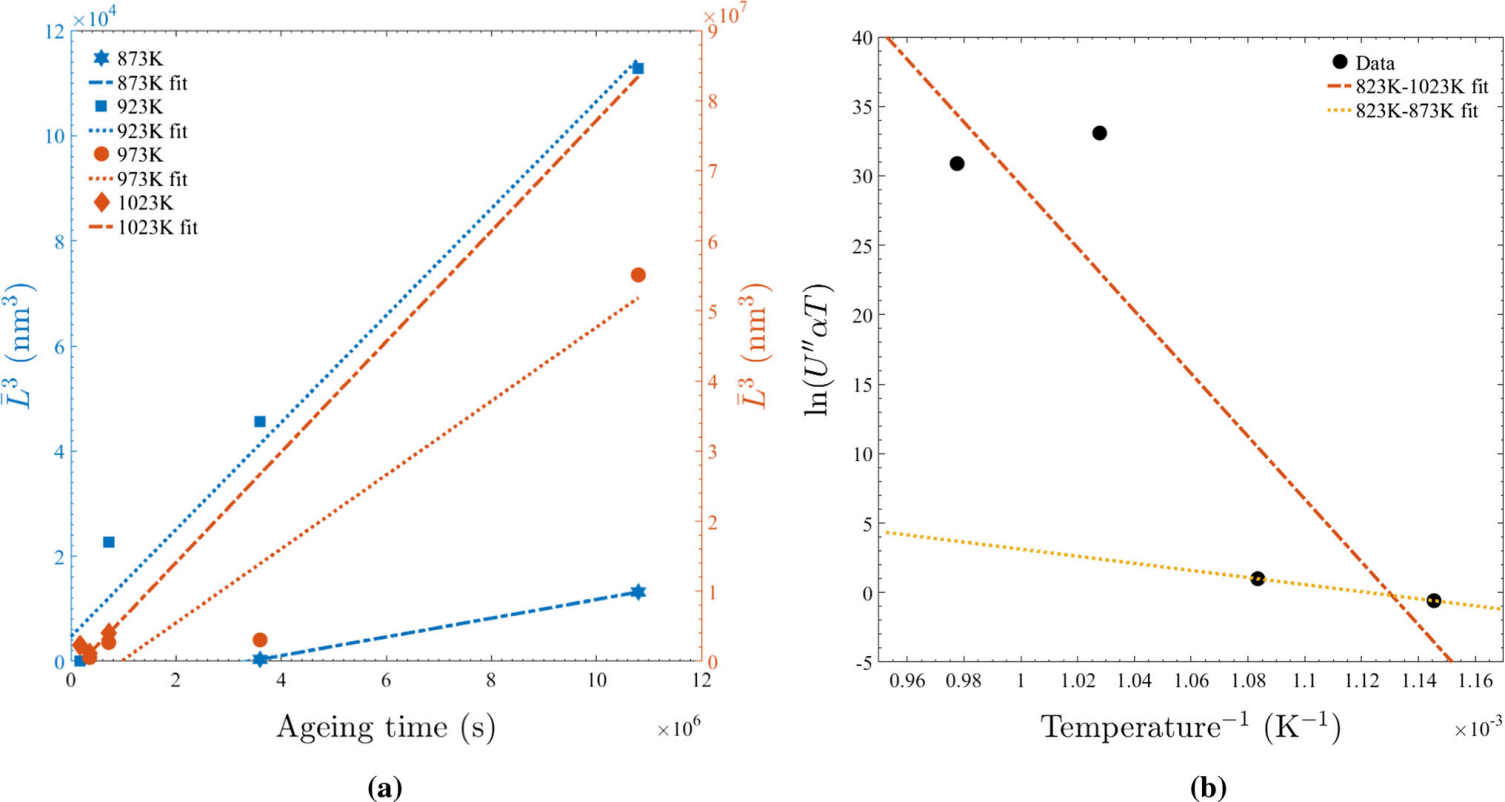
**a** $L^3$ growth behaviour of $\gamma ^{\prime \prime }$ precipitates in Alloy 625. **b** Plot to facilitate the calculation of $E_{\text {A}}$ in accordance with Eq. 2. It should be noted that whilst the spread of the data points in the 973 K series is clear evidence of a two-stage (100–200 and 1000–3000 h) precipitation behaviour, owing to the uncertainly in the start time of the homogeneous precipitation the whole series is subjected to the fit. In contrast, the wide disparity of the statistics pertaining to the heterogeneous population which exists after 50 h of ageing at 1023 K compared to the latter formed homogeneous one, and the relatively short time periods involved, means the 50 h data point is excluded from the fit in this instance

To complete the modified LSW assessment carried out in this research, the $\bar{L}_{\text {M}}^3$ growth behaviour and activation energy ($E_{\text {A}}$) for $\gamma ^{\prime \prime }$ coarsening are calculated using the methodology of previous authors [7]: through inserting an Arrhenius equation for the diffusion coefficient *D* into Eq. 1, the relationships defined in Eq. 2 are created. Consequently, the quantity $U^{\prime \prime }(T)$ can be evaluated from a linear regression to a plot of ${\bar{L}_{\text {M}}(t)}^3$ for each ageing temperature as shown in Fig. 12a. Finally, harnessing the values of $U^{\prime \prime }(T)$ and $\bar{\alpha }$ of each population, $E_{\text {A}}$ can be calculated from the gradient of a further linear regression as shown in Fig. 12b. The values of ${\bar{L}_{\text {M}}(t)}^3$ in this research corresponded to the average of between 200 and 1000 precipitates (dependent on the precipitate frequency and image magnification) measured from a number of images of material aged at each condition.

2 $$\begin{aligned} {\bar{L}_{\text {M}}^3}(t) - {\bar{L}_{\text {M}}^3}(0)\,=\, & {} U^{\prime \prime }t \nonumber \\ U^{\prime \prime }\,=\, & {} \frac{32D{_0}\zeta V_\text {m} X_\gamma ^\text {e}}{\alpha \pi RT } \frac{f_{\text {LSEM}}(\bar{L}_{\text {M}}, L^{*}_{\text {M}})^3}{C_{\text {LSEM}}}{\text {exp}\left( \frac{- E_{\text {A}}}{RT}\right) } \end{aligned}$$

The value produced from the 823–1023 K fit in Fig. 12b of $E_{\text {A}} = 1.8783\, \hbox {MJmol}^{-1}$ is far in excess of that reported by either Devaux et al. [7] (298 kJ mol$^{-1}$), Han et al. [11] ($272\, \hbox {kJ mol}^{-1}$), Wang et al. [30] ($286\,\hbox {kJ mol}^{-1}$) or Zhang et al. [31] ($292\,\hbox {kJ mol}^{-1}$) due to the significant enhancement associated with the delayed homogeneous precipitation (discussed previously) at temperatures $\ge 973\hbox {K}$. In contrast, the value of $E_{\text {A}} = 211.7\,\hbox {kJ mol}^{-1}$ calculated from the data points corresponding to samples unaffected by the transition in nucleation behaviours (873–923 K fit) is in much closer agreement with those previously found in Alloy 718. This fact is especially true when considering the decrease in $E_{\text {A}}$ that should accompany the lower temperature of the nose of the TTT curve for $\gamma ^{\prime \prime }$ in Alloy 625 [10] compared to Alloy 718 [3].

In light of the aforementioned eventualities, comparison of the results produced in this research with those of Suave et al. [24] indicates that their enhanced value of $358\,\hbox {kJ mol}^{-1}$ in Alloy 625 is attributable primarily to delayed homogeneous nucleation at 973 and 1023 K, *viz.* the two-stage behaviour in Fig. 12 (remarked upon previously) is clearly also present in their plots of $\bar{L}_{\text {M}}^3$(t). Furthermore, the cause of the effect being diminished in their work can be identified as their use of shorter ageing times and therefore the more consistent capture of a predominantly heterogeneously nucleated $\gamma ^{\prime \prime }$ precipitate population.

## Summary and conclusions

The separate, sequential key findings of this research can be summarised as follows:

1. The “directional encounter” process whereby agglomerating $\gamma ^{\prime \prime }$ coalesce along their major axis has been imaged directly through HRTEM.
2. Using the assumption of complete niobium depletion from the matrix, the LSEM description of classical precipitate growth/coarsening including the effect of “encounters” has been shown to be far more successful for $\gamma ^{\prime \prime }$ than the original LSW theory once the directional coalescence mechanism of the ellipsoidal precipitates is accounted for.
3. The shape-changing evolution of $\gamma ^{\prime \prime }$ has been demonstrated to be temperature dependent and likely consequence from the change (measured by Slama et al. [23]) induced by temperature on the misfit strain. Concomitantly, the results here find the coherence to incoherence step change suggested by Cozar et al. [5] (and latterly Devaux et al. [7]) to be a manifestation of their assumptions rather than a physical phenomenon.
4. The activation energy for $\gamma ^{\prime \prime }$ coarsening in Alloy 625 reported herein and that reported by Suave et al. [24] are in agreement when the relative precipitation mechanisms and kinetics are considered for the two studies.
5. Based on the overall results, it clear that an LSEM-type formalism will only be successful with respect to $\gamma ^{\prime \prime }$ with detailed knowledge of the behaviour of $\alpha (L_{\text {M}})$ over all relevant times and temperatures and of the relationship between “directional encounter” and $\alpha$. However, this is a significant undertaking given the effect of phases such as $\gamma ^{\prime }$ (Dong et al. [8]) and $\delta$ phase (Sundararaman et al. [27]).

## Footnotes

1 The data originally published by Dong et al. [8] were altered to account for a clear error in their values for $\bar{L}_{\text {M}}$.
